# Gathering opinion leader data for a tailored implementation intervention in secondary healthcare: a randomised trial

**DOI:** 10.1186/1471-2288-14-38

**Published:** 2014-03-10

**Authors:** Katherine Farley, Andria Hanbury, Carl Thompson

**Affiliations:** 1 Department of Health Sciences, The University of York, Seebohm Rowntree Building, York YO10 5DD, UK

## Abstract

**Background:**

Health professionals’ behaviour is a key component in compliance with evidence-based recommendations. Opinion leaders are an oft-used method of influencing such behaviours in implementation studies, but reliably and cost effectively identifying them is not straightforward. Survey and questionnaire based data collection methods have potential and carefully chosen items can – in theory – both aid identification of opinion leaders and help in the design of an implementation strategy itself. This study compares two methods of identifying opinion leaders for behaviour-change interventions.

**Methods:**

Healthcare professionals working in a single UK mental health NHS Foundation Trust were randomly allocated to one of two questionnaires. The first, slightly longer questionnaire, asked for multiple nominations of opinion leaders, with specific information about the nature of the relationship with each nominee. The second, shorter version, asked simply for a list of named “champions” but no more additional information. We compared, using Chi Square statistics, both the questionnaire response rates and the number of health professionals likely to be influenced by the opinion leaders (i.e. the “coverage” rates) for both questionnaire conditions.

**Results:**

Both questionnaire versions had low response rates: only 15% of health professionals named colleagues in the longer questionnaire and 13% in the shorter version. The opinion leaders identified by both methods had a low number of contacts (range of coverage, 2–6 each). There were no significant differences in response rates or coverage between the two identification methods.

**Conclusions:**

The low response and population coverage rates for both questionnaire versions suggest that alternative methods of identifying opinion leaders for implementation studies may be more effective. Future research should seek to identify and evaluate alternative, non-questionnaire based, methods of identifying opinion leaders in order to maximise their potential in organisational behaviour change interventions.

## Background

Healthcare delivery and outcomes vary enormously within many developed healthcare systems [1]. Even accounting for the “irreducible uncertainty” [2] associated with healthcare, much of this variation can be explained by the attitudes, clinical judgements and decisions, and consequent behaviours of health professionals [3]. One of the most powerful means of shaping these influential variables is the opinions of other healthcare professionals – particularly peers [4,5].

Particularly influential practitioners can be thought of as “opinion leaders” (OLs) [6-9]. Despite their widespread use, OLs aren’t universally effective. In a Cochrane review of 18 opinion leader studies, OLs were found to be associated with a median adjusted 12 per cent absolute increase in health professionals’ compliance with recommendations [5]. However, the range across studies varied from a 15 per cent decrease to a 72 per cent increase in compliance. The mechanisms by which they operate are only just beginning to be demonstrated and understood [10] but common features include generating consensus [11], increasing the observability and reducing the potential risk of new clinical behaviours [12], and producing more efficient learning [13-15].

Opinion leaders have been widely used in a variety of primary and community health care contexts; for example, to influence patient behaviour [13]; and as a change mechanism in health professional [16] and online [17,18] communities. In “diffusion of innovation” based theories [6,19,20], opinion leaders play a key role in increasing the uptake of recommendations and adoption of innovations - for a classic example see [21], and more recently [22-24].

### Identifying opinion leaders

Despite a range of tested techniques [13,25], identifying OLs is challenging. Methods need to be robust, reliable, attract low resources and identify high quality opinion leaders; but to be useful in implementation programmes, they also need to be able to be used alongside other research tools. Identification of OLs for use in behaviour change interventions is often informal [26], but more formal techniques used include:

● Key-informant - asking a smaller number of individuals who are knowledgeable about a network to identify influential individuals;

● Self-designating - self-reporting of own opinion leader status. This can be limiting as it does not guarantee that the OL is credible within the community and does not ensure that the OL shares the agenda of community members and researchers;

● Direct observation;

● Sociometric (analysis of leadership nominations) [19,21];

● Selection based on an individuals’ “objective” position or status, for example a celebrity or elected official [27] or someone who has published on a topic or held a key position in the organisation or geographical area [28].

Identification methods recognise the role of social networks in opinion leadership. Sociometric techniques, in which community members nominate opinion leaders, are valid, reliable and “sophisticated” (sic.) [13,15] methods of collecting opinion leader information [13]. Other methods, such as self-designating, do not always identify individuals perceived as opinion leaders by their peers. Sociometric techniques are also associated with higher response rates [26,29]. Network analysis can help identify who is most central to a community and therefore more influential [15]. All individuals are ‘actors’ within a communication network, and through the nomination of influential individuals, connections between actors can be described [30,31].

### Opinion leaders in implementation and “coverage”

A recent review of the use of social networks in healthcare implementation studies examined 52 papers [32] and revealed a growing interest in use of the technique but that the application to implementation work was limited [32]. Network data collected is often of insufficient quantity (i.e. too few contacts named) or insufficient quality (i.e. contacts identified are not suitable for use in information dissemination). Insufficient quantity can be captured and described using the ‘coverage statistic’ [6]: a rate describing the degree to which identified OLs have contact with (and, therefore, influence over) the adopter population. Coverage rates are the proportion of the (whole) population that name at least one nominated opinion leader [6]. Coverage rates are rarely and idiosyncratically reported in intervention studies [6].

Capturing more than just an OL name requires longer, and more costly, questionnaires. Typical extensions of the most basic network capture methods include multiple full name nominations of influential peers and additional information, such as the frequency of contact [33] and the direction in which the information flows between the respondent and the nominated peers [34]. Thus, the researcher seeking richer network data, with potential utility in an implementation strategy faces a trade-off: better quality data versus potentially lower response rates (longer questionnaires are less likely to be completed) and higher production and transaction costs.

### Objectives

As part of a larger implementation programme (see Hanbury et al. [35] for details of this study) to improve the provision of support for families of people with a diagnosis of psychosis. This was a three phase study. In the first, the study mapped multiple dimensions from Greenhalgh’s framework [8] as part of a diagnostic analysis to identify barriers to the use of family interventions for service-users with schizophrenia. In the subsequent implementation phase, five interventions were developed to specifically target each barrier. These interventions were an education event, promotion of outreach sessions to generate interest in the topic, promotion of the relevant clinical pathway, development of a register of co-workers). We sought to identify opinion leaders for use in an intervention to change professional behaviour. We aimed to capture good quality data without sacrificing coverage and quantity. The final phase aimed to evaluate the impact of the interventions on process of care measures [35].

Accordingly, we asked two questions:

1. Does requesting additional information about nominees affect the response rates to questionnaires collecting opinion leader information?

2. Is it possible to collect sufficient respondent ‘coverage’ rates for exploitation in implementation programmes?

Ethical approval was granted for this study by Leeds (West) Research Ethics Committee (Reference 10/H1311/1).

## Methods

### Trial design

**Figure 1:**
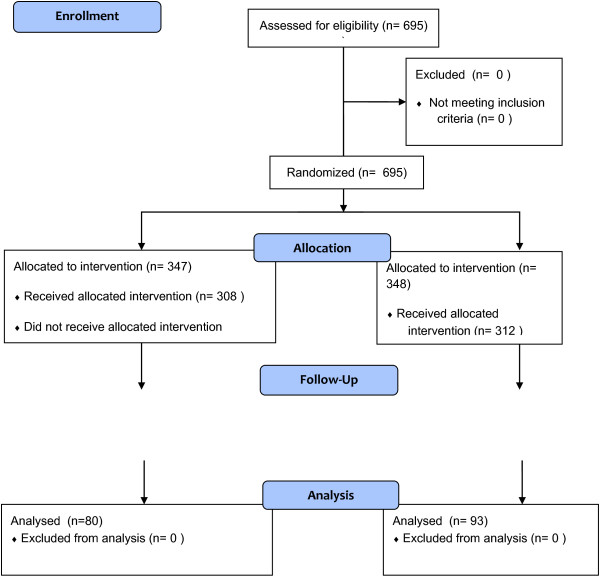
Consort diagram.

This was a randomised controlled trial design (Figure 1) of two questionnaire-based approaches to collecting opinion leader information within a larger implementation study. The trial took place in a single, large (~4000 staff), mental health and learning disability NHS Foundation Trust in the North of England.

### Participants

All healthcare professionals in the NHS Trust involved in the care and management of patients with a psychosis diagnosis were eligible to participate. The Trust’s Medical Director provided the names of eligible staff. See Table 1 for details of participants.

**Table 1:** Population characteristics

	**Long variant**		**Short variant**		**Total %**
**Job role**	**N /347**	**% of variant**	**N/348**	**% of variant**	
Clinical psychologist	19	5.4	14	4	4.74
Community nurse	26	7.5	20	5.8	6.6
Healthcare assistant/Healthcare support worker	7	2	8	2.3	2.15
Modern matron	2	0.6	5	1.44	1.0
Sister/Charge nurse	32	9.2	32	9.2	9.2
Staff nurse	136	39	138	40	39.4
Psychotherapist	11	3.2	8	2.3	2.73
Occupational therapist	48	14	42	12	12.9
Midwife	0	0	2	0.57	0.29
Nurse manager	14	4	25	7.2	5.6
Nurse consultant	1	0.28	0		0.14
Community practitioner	48	14	49	14	13.9
Senior nurse practitioner	2	0.6	4	1.1	0.86
Other	1	0.28	1	0.28	0.29

#### The intervention

The “intervention” took the form of one of two differing OL identification questions embedded in otherwise identical questionnaires:

a) The first ‘sociometric’ variant (Additional file 1) asked respondents to nominate individuals with whom they discussed the topic of schizophrenia over the past twelve months. Respondents were asked to provide names and job roles of their nominated contacts as well as the ‘direction’ of contact (who usually gives or receives advice) and the frequency and mode of communication. We planned to use this information to help design methods to influence attitudes, norms and intentions and eventually, professional behaviour.

b) Variant 2 – the ‘*Brief’* nomination tool (Additional file 2): respondents were asked to name anyone they perceived to ‘strongly influence’ local practice in the area of schizophrenia. The only subsequent information requested was the contact’s job role and whether they part of the respondent’s own team. We asked the team question to identify OLs whose influence extended between teams and across formal structures.

### Outcomes

The primary outcome was the, successfully completed questionnaire, response rate in each intervention arm. Rates were calculated from all questionnaires received at the end of April 2011. The secondary outcome measure was the coverage rate in each questionnaire variant. The formula developed by Grimshaw and colleagues [6] were used to calculate coverage. No changes were made to these outcome measures after the trial commenced.

### Data collection, sample size and randomisation

Questionnaires were administered to 695 individuals during March and April 2011. Participants were randomly allocated to either the sociometric or the brief version of the questionnaire using simple randomisation via the RAND function in excel. To maximise response rates, participants were given a choice of paper and online (accessed via a hyperlink in an email) versions of both questionnaires. Individualised pre-notification letters were sent [36]. A reminder email and link to the online questionnaire was sent two weeks later to those who had not responded. Three weeks later a paper based reminder with a paper copy of the questionnaire was sent to all remaining non-responders. We marketed the survey via the NHS Trusts’ electronic newsletter, postcard-sized promotion leaflets, and senior managers in the Trust were asked to encourage colleagues to participate in meetings and committees.

### Analysis

Response rates for each questionnaire version were calculated. We calculated separate rates for the questionnaire minus the OL identification section and for the OL section alone for each questionnaire. A list of contacts nominated by respondents was generated, to which we applied the definition of ‘Opinion Leader’ employed by Grimshaw et al. [6]: an individual nominated by more than one respondent [6,37]. This created a distinction between ‘contacts’ named by one respondent only and ‘Opinion Leaders’ nominated by more than one respondent.

Analysis had four main stages:

1. Response rates for each questionnaire version were calculated and compared using chi square, non parametric tests of significance.

2. Respondent coverage rates were calculated: the percentage of respondents linked to a nominated OL. The coverage rate was the percentage of survey respondents nominating *at least one* of the OLs. Double counting was prevented by not counting cases where an individual had nominated more than one OL.

3. Population coverage rates for OLs were also calculated [6]: the proportion of the population linked to an OL. This was calculated by dividing this number by the total survey population (n = 695).

4. Maximum coverage rate for any single OL was the last to be calculated: the individual opinion leader nominated by the most respondents. Coverage rates were calculated for each questionnaire version separately and then combined.

## Results

### Recruitment

75 questionnaires were returned as staff had left the NHS Trust. A qualitative research study undertaken after the trial indicated that the Trust’s own human resources data was of poor quality and thus the amount of incorrect contacts in the sampling frame may be considerably higher. Table 2 indicates the characteristics of respondents included in analysis.

**Table 2:** Respondent characteristics/numbers analysed

	**Long variant**		**Short variant**	
	**Response to survey**	**Response to opinion leader section**	**Response to survey**	**Response to opinion leader section**
Sister	10	3	9	2
Occupational therapist	12	6	14	2
Staff nurse	11	5	23	6
Enrolled nurse	1	1	0	
Community nurse	6	5	6	2
Community practitioner	13	6	14	3
Nurse manager	4	1	6	3
Clinical psychologist	3	0	6	3
Nurse consultant	1	1	0	0
Community health worker	0	0	1	0
Specialist nurse practitioner	0	0	1	0
Nurse manager	1	0	1	0
Psychotherapist	4	0	5	2
No job role provided	14	17	7	16

### Respondent characteristics/Numbers analysed

#### Primary outcome: the response rate

Response rates were not significantly [(1, N = 173), =1.36, *p* .244] higher for the brief version (30%, N = 93) than for the sociometric version (25%, N = 80). There was also no statistically significant difference between the sociometric and the brief questionnaire in responses to the OL section specifically, or the number of respondents providing at least one full name. More full names were provided by respondents to the longer version than by the single item technique, but not significantly so (Table 3).

**Table 3:** Sample and response rates

	**Sociometric version (N = 348)**	**Brief version (N = 347)**	**Chi sq value**	**Asymp.sig.**
**Survey response rate**	26% (80)	30% (93)	1.355	.244
**Section response rate**	15% of population 45	13% of population 39	.496	.481
**Response rate of respondents providing at least one name**	9.7% of population 30	5% of population 16	3.252	.071
**Number of contact names provided**	89	43		

C***overage:*** Table 4 provides coverage rates for each variant. Thirteen individuals met the criteria for opinion leaders (more than one nomination). Three were identified by the brief questionnaire, three were identified via the sociometric questionnaire and a further seven OLs were nominated by staff completing both versions. The percentage of respondents linked via these OLs (respondent coverage) was calculated for each version separately (of these opinion leaders, seven were nominated by just two respondents and the remaining six were named by three or more respondents). For the brief survey the respondent coverage rate was 13.3% and the sociometric, 11.25%, making the total respondent coverage rate just 12.14%. The population coverage rate for the short survey was 3.85% and for the long 2.9%. The maximum coverage rate of a single OL was 5%.

**Table 4:** Coverage rates

	**Sociometric survey**	**Brief survey**	**Total**
**Number of OLs (identified by >1 respondent)**	10 (of which 7 also named in brief version)	10 (of which 7 also named in sociometric version) (4 named both inside and outside team)	13
**Respondent coverage**	9 (11.25%)	12 (13.3%)	21 (12.14%)
**Population coverage**	9 (2.9%)	12 (3.85%)	21 (3.02%)

Respondent coverage is the proportion of those who responded to the survey who named at least one of the nominated opinion leaders. Population coverage is the proportion of the whole population who named at least one of the nominated opinion leaders.

## Discussion

Comparing two questionnaires, we found that response rates to questions about opinion leaders did not differ regardless of the approach used. Our second finding was that the opinion leaders identified via these questionnaires reached only a small proportion of the population. These results reflect those of Grimshaw and colleagues [6] but coverage is far lower than those of Cosens et al. [29] who achieved a 58% population coverage rate. With low response rates, it is unsurprising that population coverage rates were also very low. Questionnaire length and the amount of information requested did not affect response rates. Optimistically, this means that implementation strategy designers seeking to identify opinion leaders *and* ask for information of value to designing behaviour change interventions should feel comfortable doing so. The value that this additional information can add to implementation may be worthwhile if this approach does not incur any extra cost.

Less optimistically however, response rates were low to *both* approaches. Low response rates may be attributed to the length of the questionnaire overall: that our response rate was lower than some similar studies (Doumit et al., [37] achieved a 38% response rate whilst Cosens et al. [29] achieved 61%) may be due to the opinion leader section forming just one component of a longer questionnaire exploring multiple determinants of innovation adoption. Questionnaire approaches that focus *only* on gathering opinion leader data may achieve higher response rates, for example, Cosens et al. [29] achieved 40% response rate to the opinion leader section in their study. We hypothesise that these better response rates may be due to the reduced burden on participants. If implementation intervention designers are wedded to questionnaire based approaches to OL identification then this trade-off between the need to reduce burden and simultaneously collect data on several factors (such as attitudes, norms and intentions, as well as OL nominations) will be unavoidable. Those health professionals who were motivated to respond to the questionnaire were less willing to respond to questions about their contacts; a pattern also identified by Grimshaw and colleagues, who found some respondents perceived the notion of opinion leaders as too “abstract”, making OL identification questions difficult to answer [6]. A reluctance to provide names may present significant challenge to collecting opinion leader information from community nominations, regardless of the techniques used.

Increased response rates with questionnaires could be possible [36]. Whilst proven techniques were applied in this study (for example, an incentive of entry into a prize draw was offered to all respondents, respondents had the opportunity to complete either a paper or online copy of the survey, reminder emails and letters were sent to non-respondents) we still had a poor response. It is likely then that the questionnaire length together with the reluctance to name individual colleagues reduced response rates.

These findings should lead implementation experts to ask whether gathering opinion leader information using questionnaires is a suitable approach; especially given the burden placed on respondents by multidimensional questionnaires. Social networks and opinion leaders operate within a wider implementation context [38]. By focussing on systematically collecting questionnaire based opinion leader data this wider context may be missed. Whilst individual actors and organisational context are both important, it is increasingly recognised that the interplay between these is critical [38]. Face to face collection of social network data permits a better understanding of the context in which peer communication takes place and of the precise nature of the communication and relationships.

Low response and coverage rates mean that data on opinion leaders cannot be usefully applied to implementation efforts. Even with high quality data and high coverage rates, subsequent utilisation of opinion leaders in implementation strategies is challenging. For example, administrative delays in implementation work can make it difficult to harness the support of opinion leaders [11]; identifying individuals is not sufficient if they do not endorse the innovation [26], those identified may not view the innovation to be adopted positively [6], and the opinion leader status may be temporary [37]. Thus, the implementation designer must face the challenge of collecting sufficient information about opinion leaders; which in turn requires a more comprehensive tool and thus induces greater sense of burden in respondents.

### Limitations

The Trust’s own contact list was of out of date and inaccurate, this seriously hampered the construction of a good quality sampling frame. As an external team of researchers we were reliant upon the information provided by the NHS Trust. Our misplaced assumption that this information was comprehensive and up-to-date meant that our questionnaire did not reach a proportion of the population which in turn affected our sample size and response rates.

We did not collect economic data on the time and financial resources consumed by both approaches. This was an omission and prevented a more informed assessment of the relative cost effectiveness of each approach.

The study took place within a changing organisational context, which may have influenced response rates to the questionnaire, and makes generalising these findings to more stable organisations difficult. The relationship between response rates and coverage rates is unknown: higher response rates may have generated different coverage rates. Lastly, using a questionnaire that focused solely on gathering opinion leader data may have provided better response rates.

## Conclusions

Theoretically and empirically, informal communication channels are a valuable means of diffusing innovation [12,19,20,37]. However, collecting sufficient information using social network techniques is resource intensive and requires high levels of engagement from both researcher and respondents. Our results suggest that questionnaire based approaches to OL identification lead to information that is almost unusable in the context of designing a theoretically-informed behaviour change intervention. The study reinforces Grimshaw and colleagues’ [6] assertion that there is limited empirical evidence to support the collection of opinion leader data. Other researchers may wish to consider future studies which compare, and assess the cost-effectiveness of, OL-only questionnaires vs. embedded approaches, or questionnaire vs. qualitative/observational techniques.

## Competing interests

The authors declare that they have no competing interests.

## Authors’ contributions

KF participated in the design of the project protocol, developed the questionnaire, conducted the analysis presented here, and drafted the manuscript. AH designed the project protocol, developed the questionnaire, and drafted the manuscript. CT designed the project protocol, developed the questionnaire and reviewed the manuscript. All authors read and approved the final manuscript.

## Pre-publication history

The pre-publication history for this paper can be accessed here:

http://www.biomedcentral.com/1471-2288/14/38/prepub

## Supplementary Material

Additional file 1 Questionnaire variant 1. Click here for file

Additional file 2 Questionnaire variant 2. Click here for file
