# Supplementary material for: Gathering opinion leader data for a tailored implementation intervention in secondary healthcare: a randomised trial
Source: BMC Med Res Methodol. 2014 Mar 10;14:38. doi: 10.1186/1471-2288-14-38 (PMC4015818; doi:10.1186/1471-2288-14-38)
Supplement: Additional file 1 — Questionnaire variant 1. [file 1471-2288-14-38-S1.pdf]

Additional File 1

Section Two **Communication Networks**

In this section of the survey we are interested in the people you have had contact with over the past 12 months about your day-to-day practice in the management of schizophrenia. We ask for this information because sharing information with colleagues is seen as a way of spreading good practice and diffusing new ideas. These may be people from whom you have sought guidance or information, people who have sought guidance or information from you, or simply people with whom you have generally discussed practice in this area. We have provided room for a number of people's names, however, do not feel that you have to fill all of the boxes.

Which members of your immediate team, with whom you work day-to-day, have you sought advice from, or given advice to on the management of schizophrenia?

19 **Full name of Person 1**...

How often have you discussed this topic with them? (please circle)

once a month	once a week	several times a week

Do you usually

seek advice from them	provide advice	Both provide and seek advice

What is their job role?

…………………………………………………………………………………….

20 **Full name of Person 2**...

How often have you discussed this topic with them?

once a month	once a week	several times a week

Do you usually

seek advice from them	provide advice	Both provide and seek advice

What is their job role?

…………………………………………………………………………………….

21 Full name of Person 3...

How often have you discussed this topic with them?

once a month	once a week	several times a week

Do you usually

seek advice from them	provide advice	Both provide and seek advice

What is their job role?

…………………………………………………………………………………………….

22 Full name of Person 4...

How often have you discussed this topic with them?

once a month	once a week	several times a week

Do you usually

seek advice from them	provide advice	Both provide and seek advice

What is their job role?

…………………………………………………………………………………………….
