# Supplementary material for: Gathering opinion leader data for a tailored implementation intervention in secondary healthcare: a randomised trial
Source: BMC Med Res Methodol. 2014 Mar 10;14:38. doi: 10.1186/1471-2288-14-38 (PMC4015818; doi:10.1186/1471-2288-14-38)
Supplement: Additional file 2 — Questionnaire variant 2. [file 1471-2288-14-38-S2.pdf]

Additional file 2

Section Two Communication Networks

In this section we are interested in people who are seen locally as having a significant influence on how service-users with schizophrenia are managed in the area.

19 **Are there any people within your team who you feel strongly influence local practice in the field of schizophrenia management?**

Please give their names and job roles

……………………………………………………………………………………………
.

……………………………………………………………………………………………

……………………………………………………………………………………………

……………………………………………………………………………………………

20 **Are there any people outside of your team who you think are seen locally as 'leaders' in the field of schizophrenia management?**

Please give their names and job roles

……………………………………………………………………………………………
.

……………………………………………………………………………………………

……………………………………………………………………………………………

……………………………………………………………………………………………
